# Supplementary material for: Deep Learning‐Based Accelerated MR Cholangiopancreatography Without Fully‐Sampled Data
Source: NMR Biomed. 2025 Feb 5;38(3):e70002. doi: 10.1002/nbm.70002 (PMC11795733; doi:10.1002/nbm.70002)
Supplement: Supplementary file 1 — Figure S1: Results of retrospective undersampling at 3 T for the volunteer #1. Figure S2: Results of retrospective undersampling at 3 T for the volunteer #2. Figure S3: Results of retrospective undersampling at 3 T for the volunteer #3. Figure S4: Results of retrospective undersampling at 3 T for the volunteer #4. Figure S5: Results of retrospective undersampling at 3 T for the volunteer #5. Figure S6: Results of retrospective undersampling at 3 T for the volunteer #6. Figure S7: Results of retrospective undersampling at 3 T for the volunteer #7. Figure S8: Results of retrospective undersampling at 3 T for the volunteer #8. Figure S9: Results of retrospective undersampling at 0.55 T for the volunteer #9. Figure S10: Results of retrospective undersampling at 0.55 T for the volunteer #10. Figure S11: Results of retrospective undersampling at 0.55 T for the volunteer #11. Figure S12: Results of prospective undersampling at 3 T for the volunteer #1. Figure S13: Results of prospective undersampling at 3 T for the volunteer #2. Figure S14: Results of prospective undersampling at 3 T for the volunteer #3. Figure S15: Results of prospective undersampling at 3 T for the volunteer #4. Figure S16: Results of prospective undersampling at 3 T for the volunteer #5. Figure S17: Results of prospective undersampling at 3 T for the volunteer #6. Figure S18: Results of prospective undersampling at 3 T for the volunteer #7. Figure S19: Results of prospective undersampling at 3 T for the volunteer #8. Figure S20: Results of prospective undersampling at 0.55 T for the volunteer #9. Figure S21: Results of prospective undersampling at 0.55 T for the volunteer #10. Figure S22: Results of prospective undersampling at 0.55 T for the volunteer #11. [file NBM-38-e70002-s001.docx]

Supporting Information

**Deep Learning-based Accelerated MR Cholangiopancreatography without Fully-sampled Data**

Jinho Kim ^1,2,*^, Marcel Dominik Nickel ^2^, Florian Knoll ^1,3^

^1^Department of Artificial Intelligence in Biomedical Engineering, Friedrich-Alexander-Universität Erlangen-Nürnberg, Germany

^2^Research & Clinical Translation, Magnetic Resonance, Siemens Healthineers AG, Erlangen, Germany

^3^Center for Advanced Imaging Innovation and Research (CAI2R), Department of Radiology, New York University Grossman School of Medicine, New York, NY, USA

* Correspondence can be addressed to J.K. (jinho.kim@fau.de)

**CONTENTS**

**Supplemental Figure 1-8:** Results of retrospective undersampling at 3T for the volunteer #1-8

**Supplemental Figure 9-11:** Results of retrospective undersampling at 0.55T for the volunteer #9-11

**Supplemental Figure 12-19:** Results of prospective undersampling at 3T for the volunteer #1-8

**Supplemental Figure 20-22:** Results of prospective undersampling at 0.55T for the volunteer #9-11

**
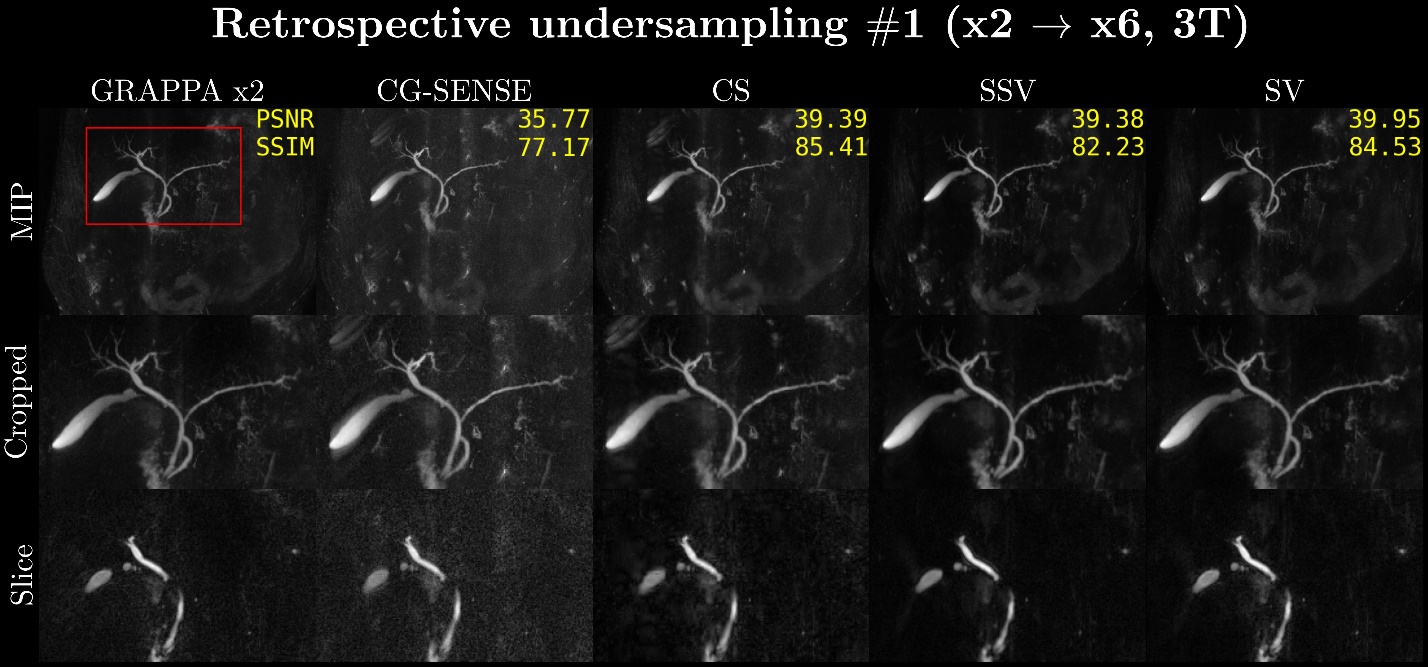
**

**Supplemental Figure 1:** Results of retrospective undersampling at 3T for the volunteer #1

**
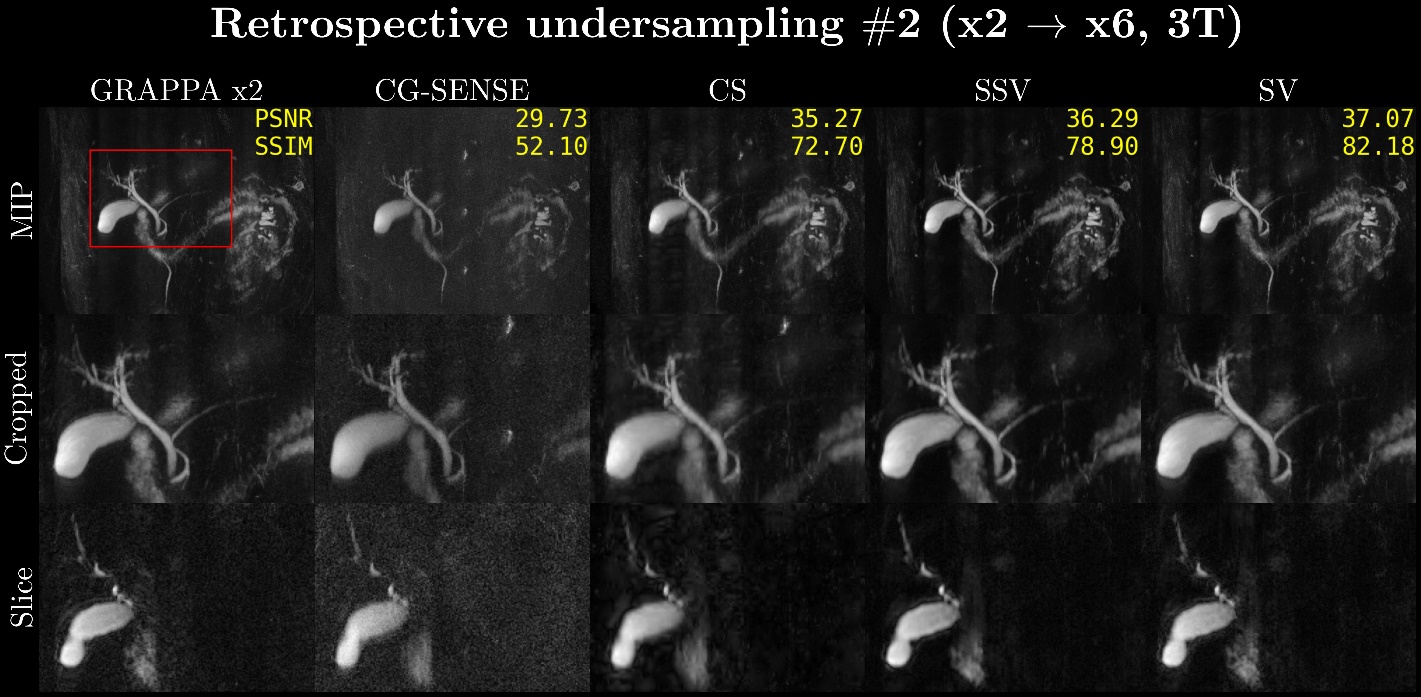
**

**Supplermental Figure 2:** Results of retrospective undersampling at 3T for the volunteer #2

**
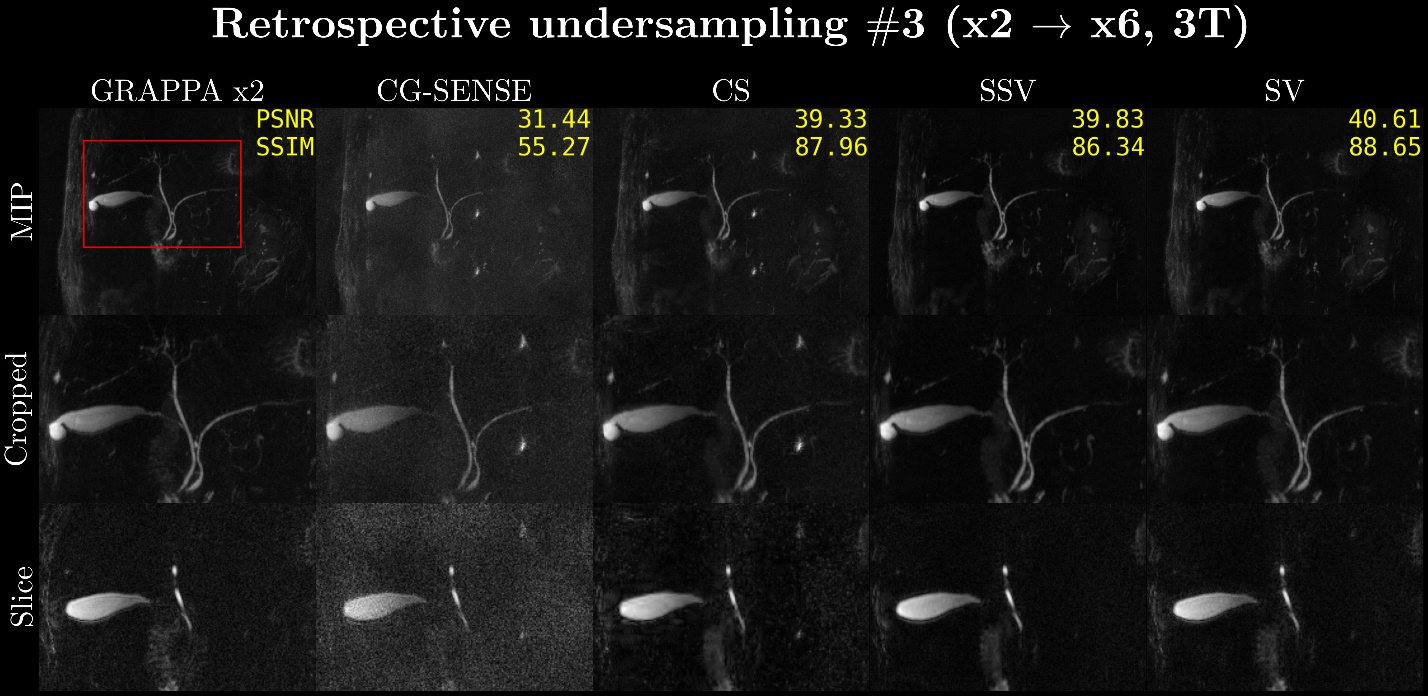
**

**Supplemental Figure 3:** Results of retrospective undersampling at 3T for the volunteer #3

**
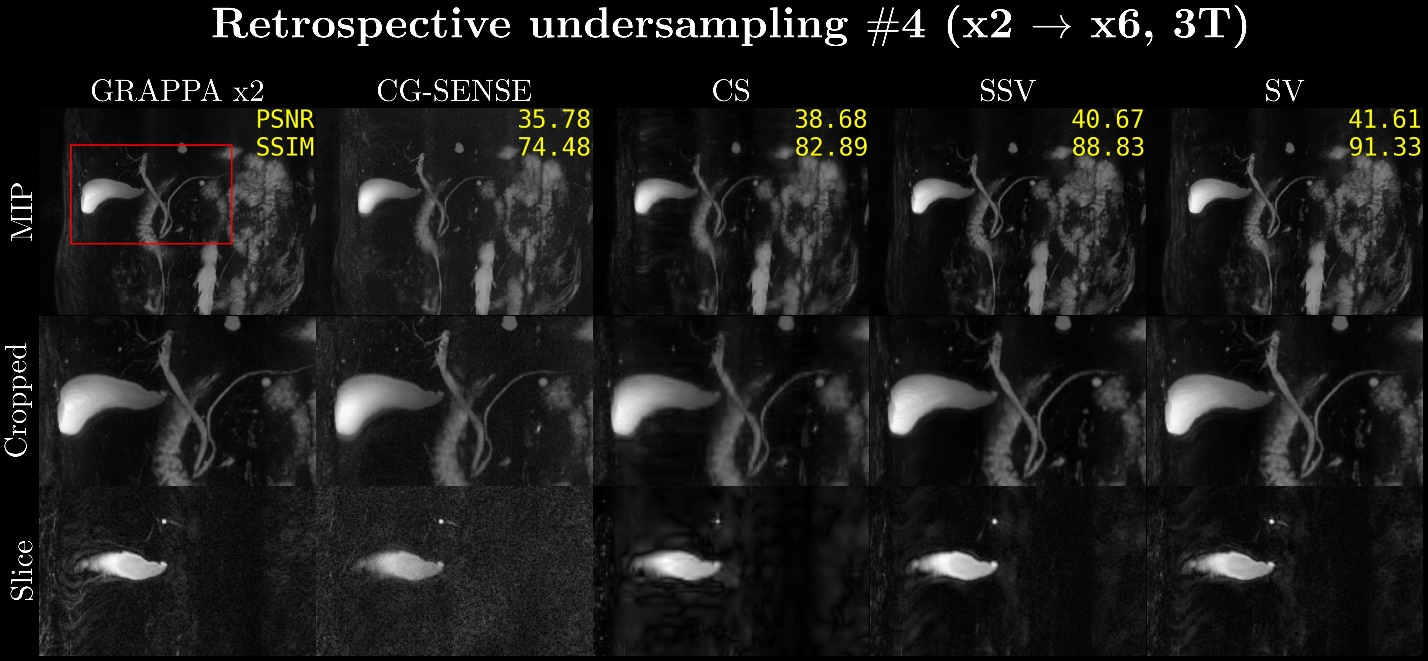
**

**Supplemental Figure 4:** Results of retrospective undersampling at 3T for the volunteer #4

**
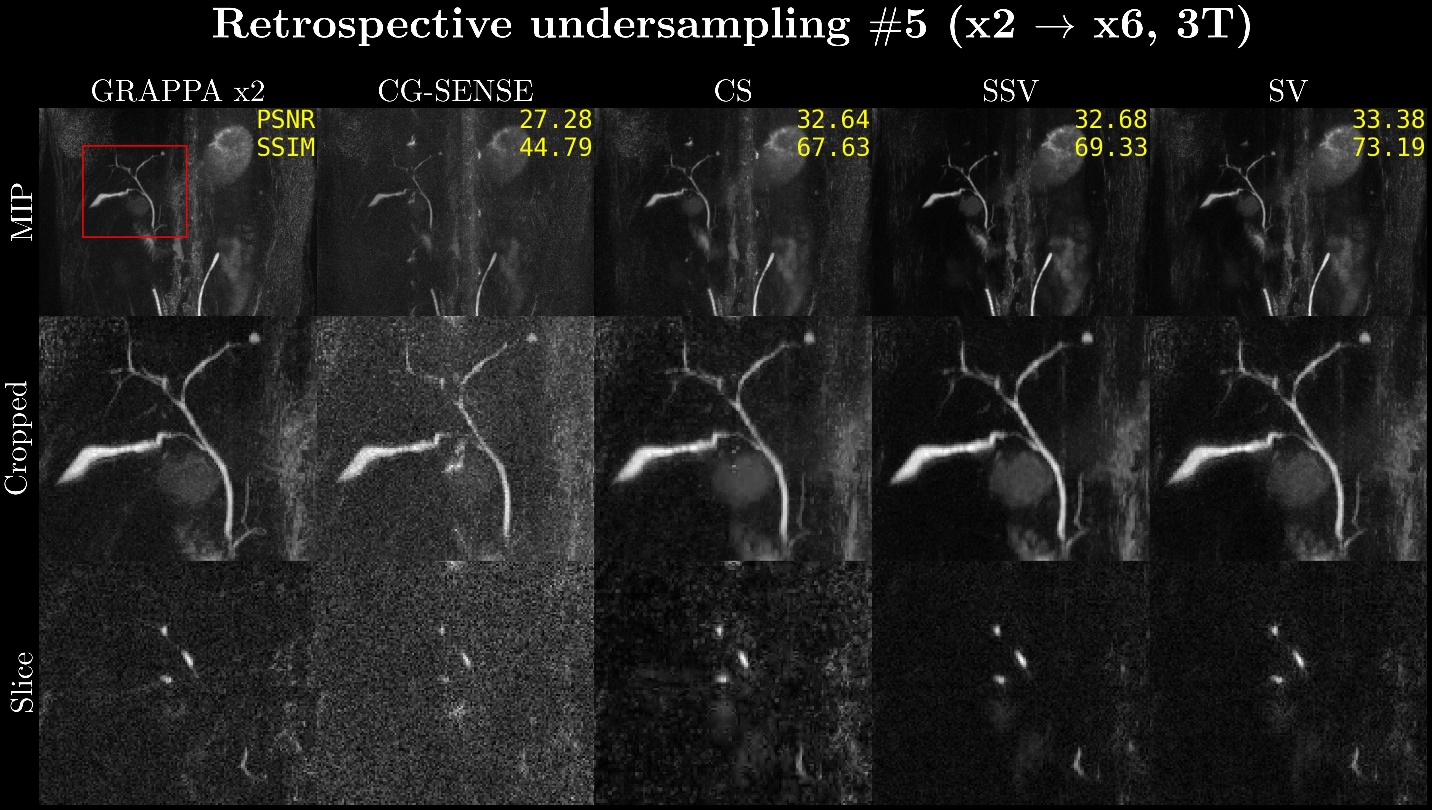
**

**Supplemental Figure 5:** Results of retrospective undersampling at 3T for the volunteer #5

**
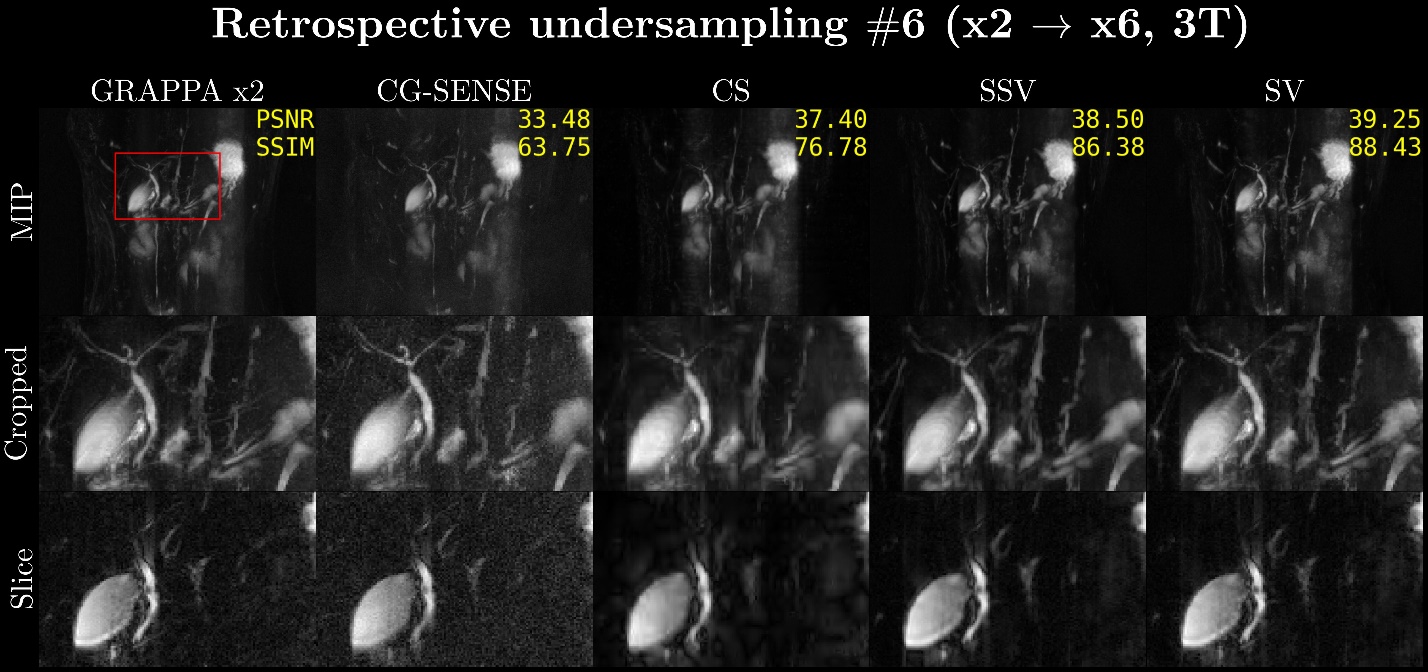
**

**Supplemental Figure 6:** Results of retrospective undersampling at 3T for the volunteer #6

**
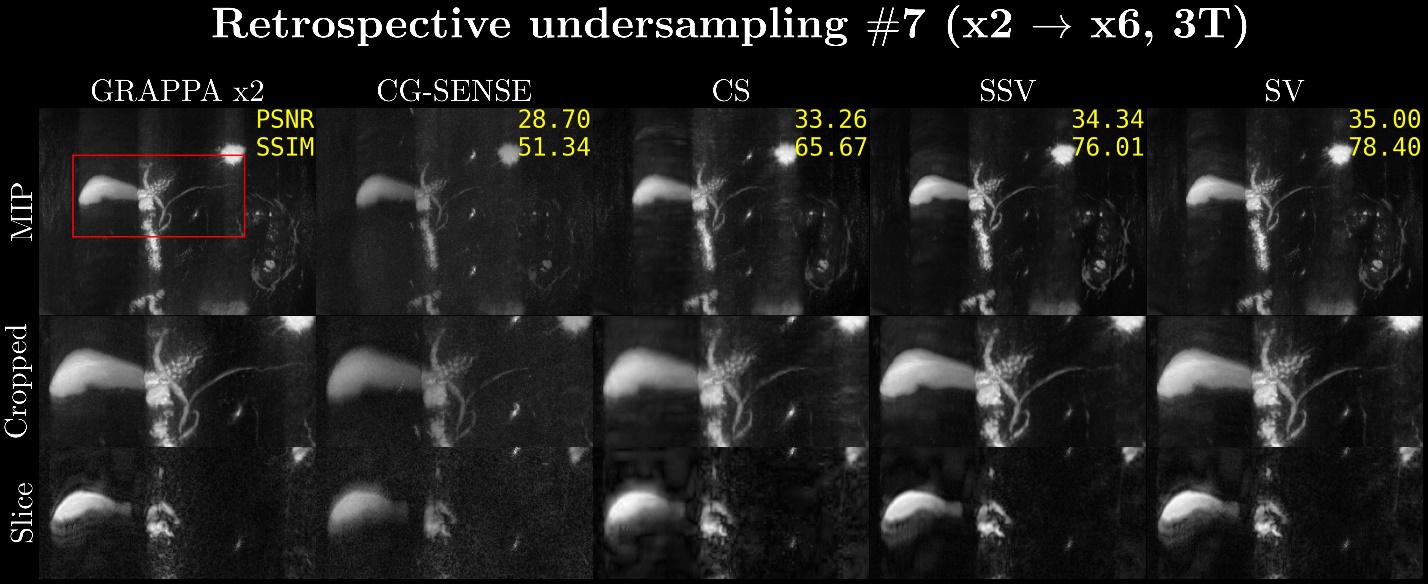
**

**Supplemental Figure 7:** Results of retrospective undersampling at 3T for the volunteer #7

**
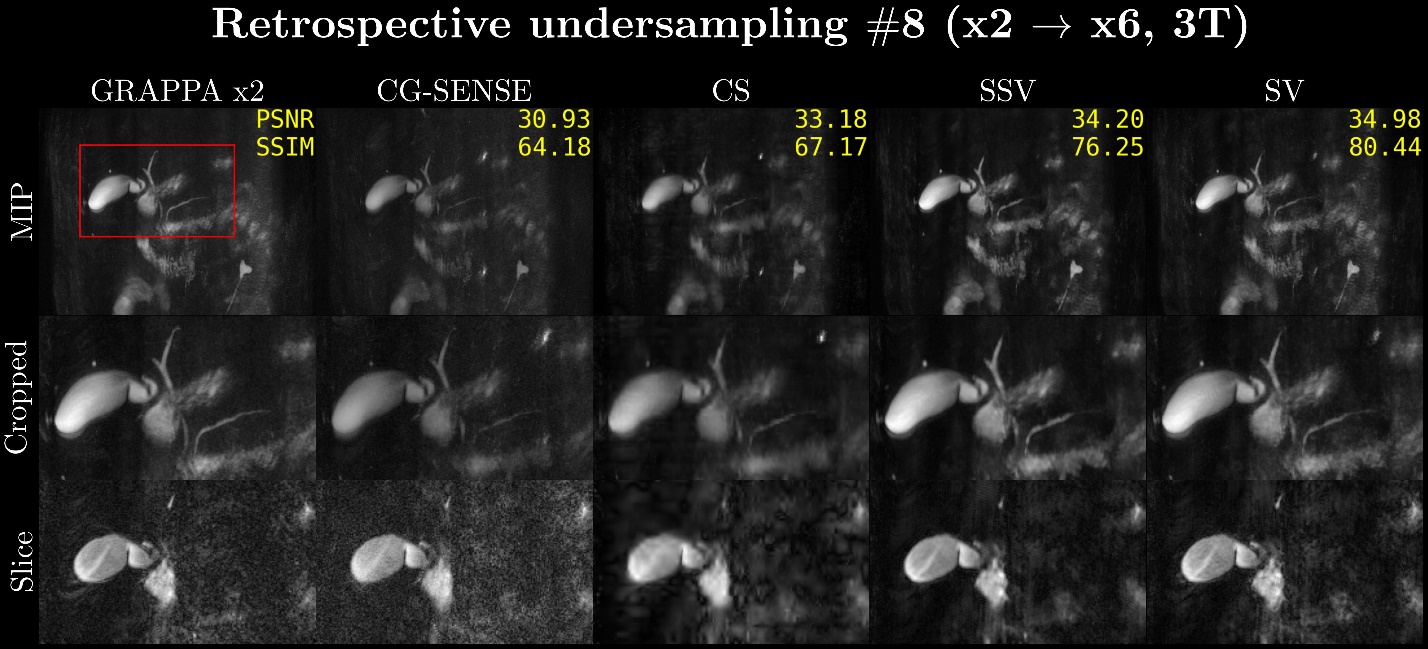
**

**Supplemental Figure 8:** Results of retrospective undersampling at 3T for the volunteer #8

**
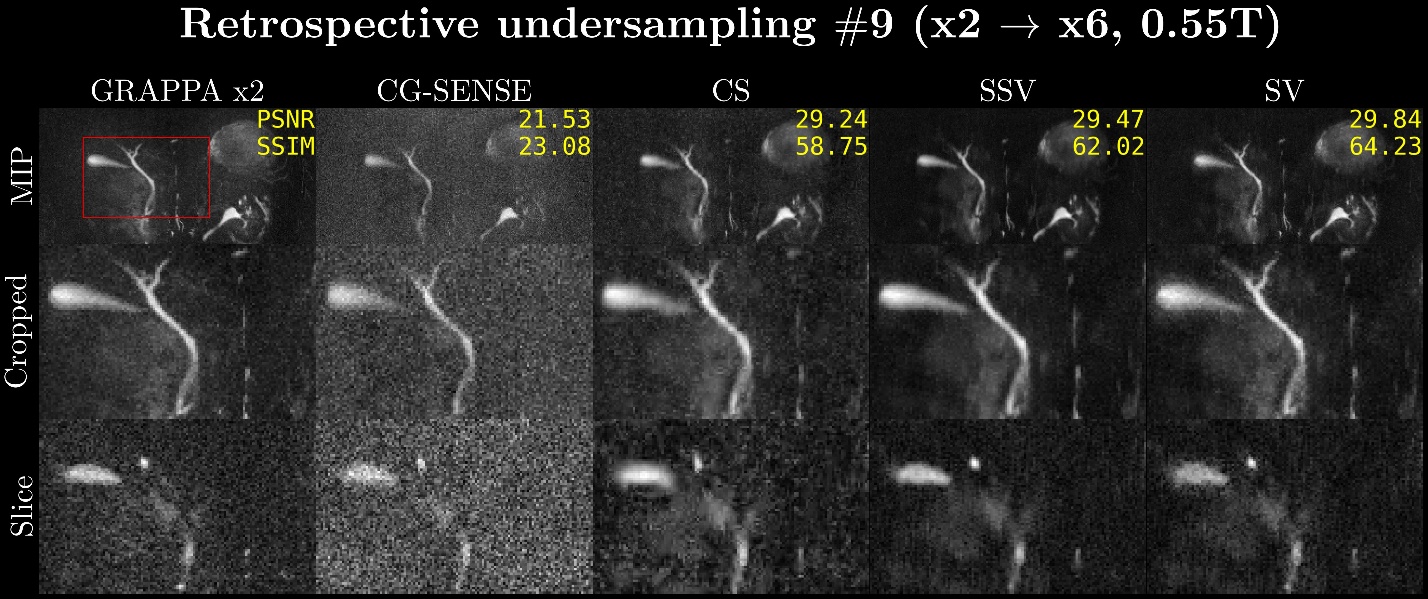
**

**Supplemental Figure 9:** Results of retrospective undersampling at 0.55T for the volunteer #9

**
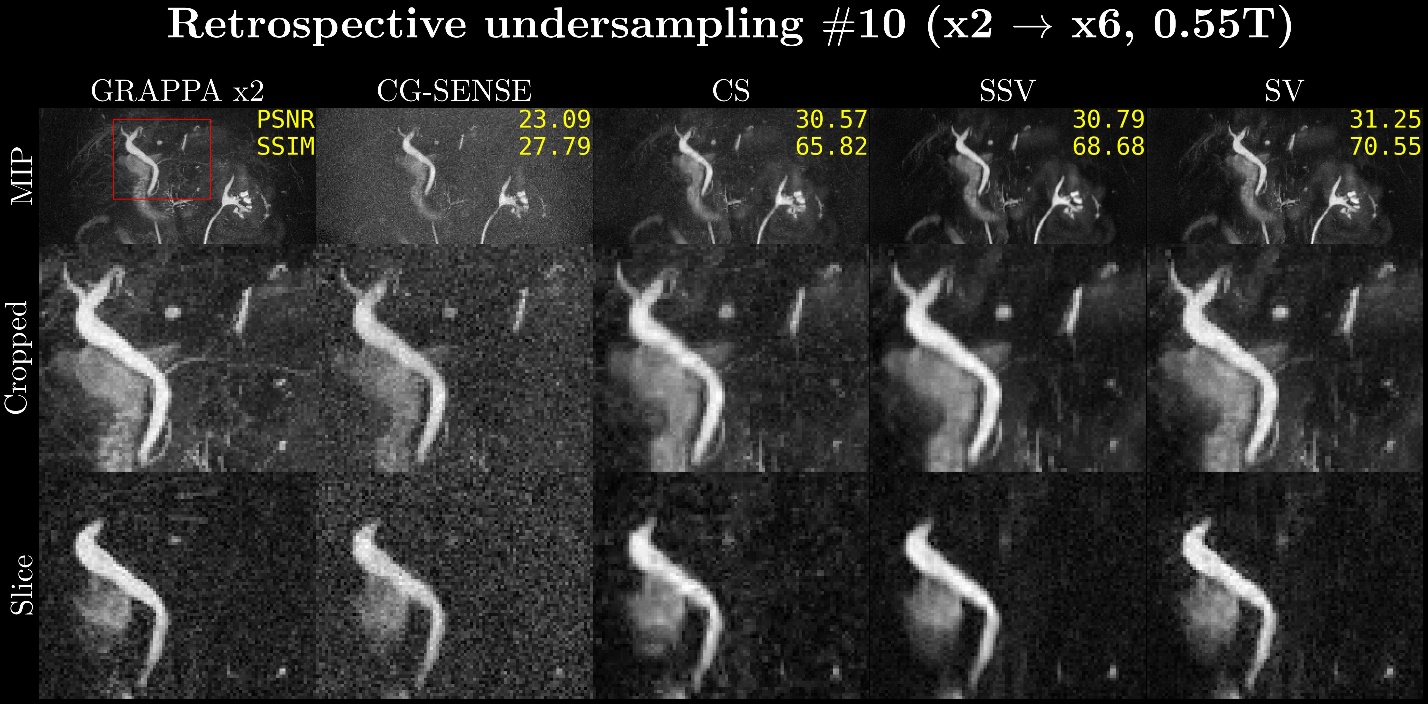
**

**Supplemental Figure 10:** Results of retrospective undersampling at 0.55T for the volunteer #10

**
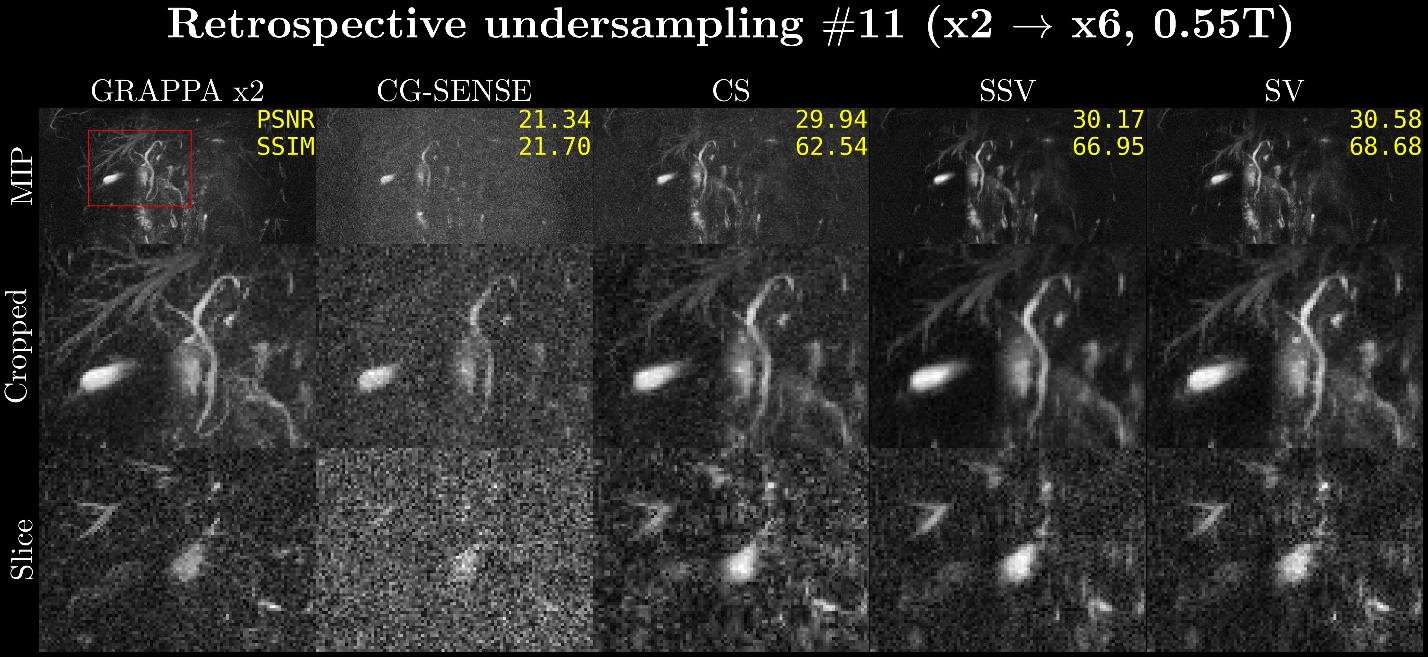
**

**Supplemental Figure 11:** Results of retrospective undersampling at 0.55T for the volunteer #11

**
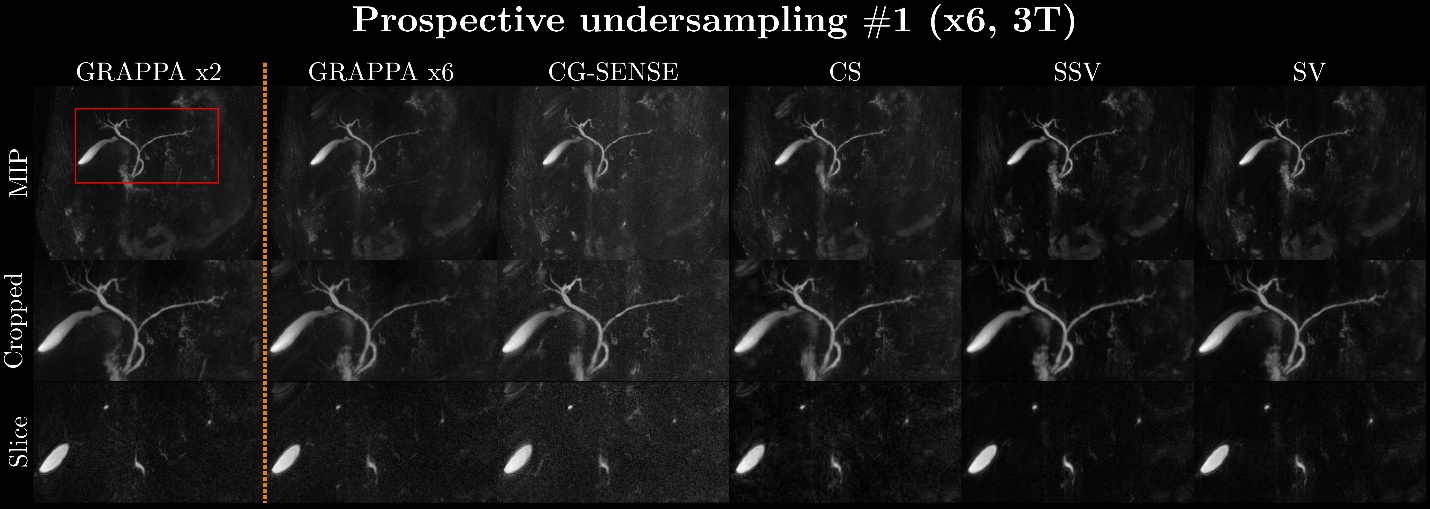
**

**Supplemental Figure 12:** Results of prospective undersampling at 3T for the volunteer #1

**
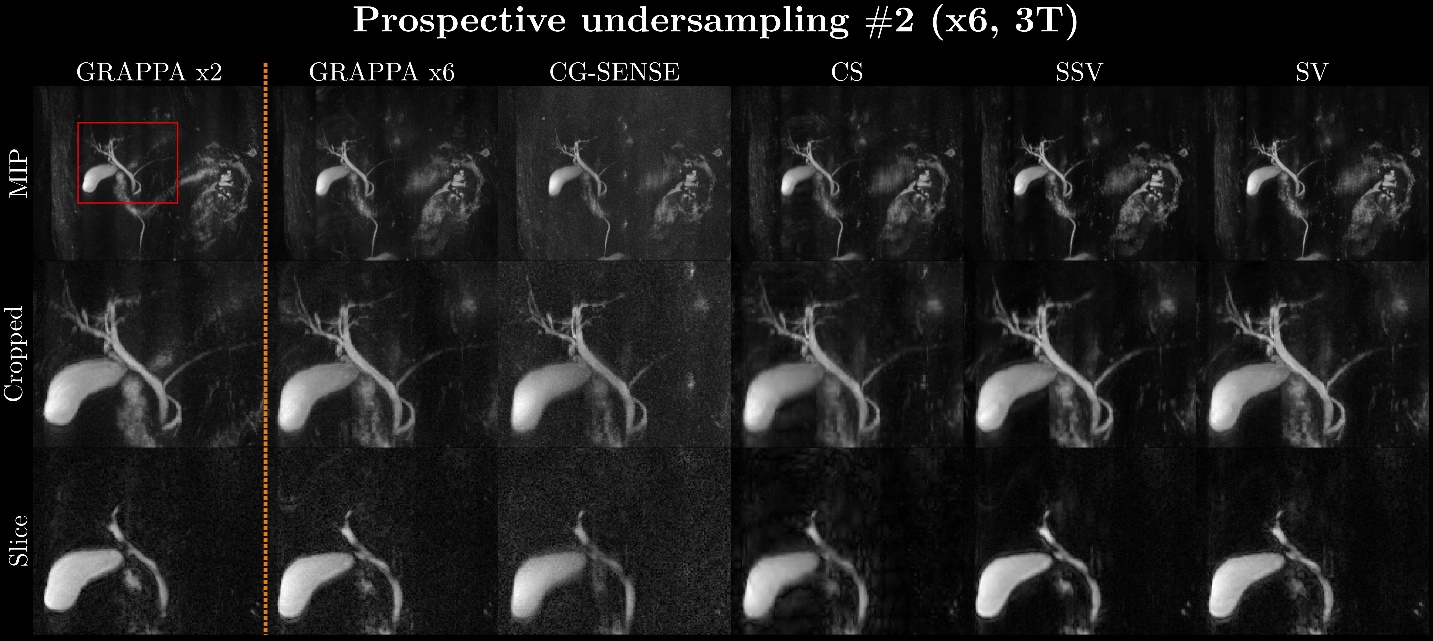
**

**Supplemental Figure 13:** Results of prospective undersampling at 3T for the volunteer #2

**
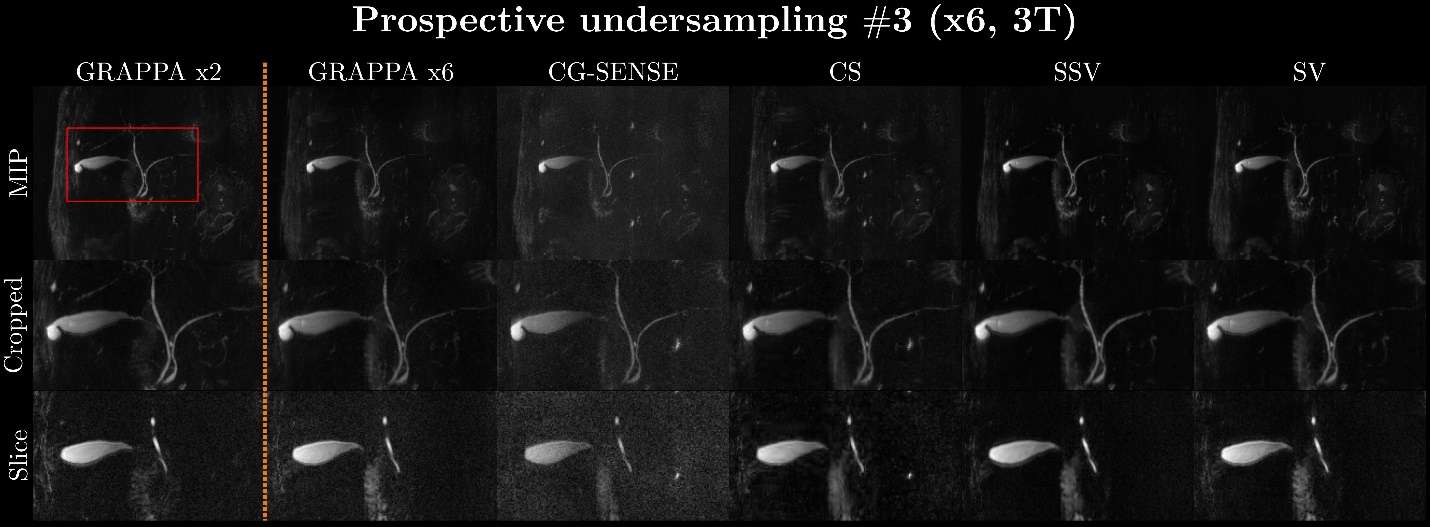
**

**Supplemental Figure 14:** Results of prospective undersampling at 3T for the volunteer #3

**
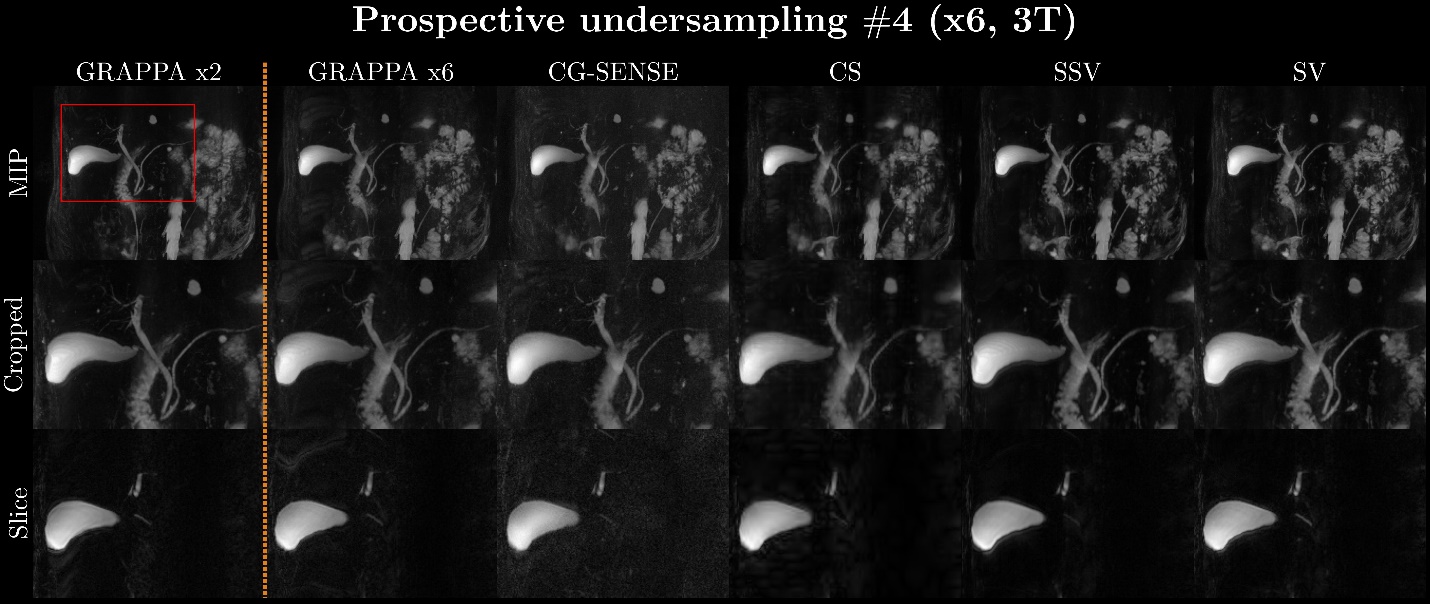
**

**Supplemental Figure 15:** Results of prospective undersampling at 3T for the volunteer #4

**
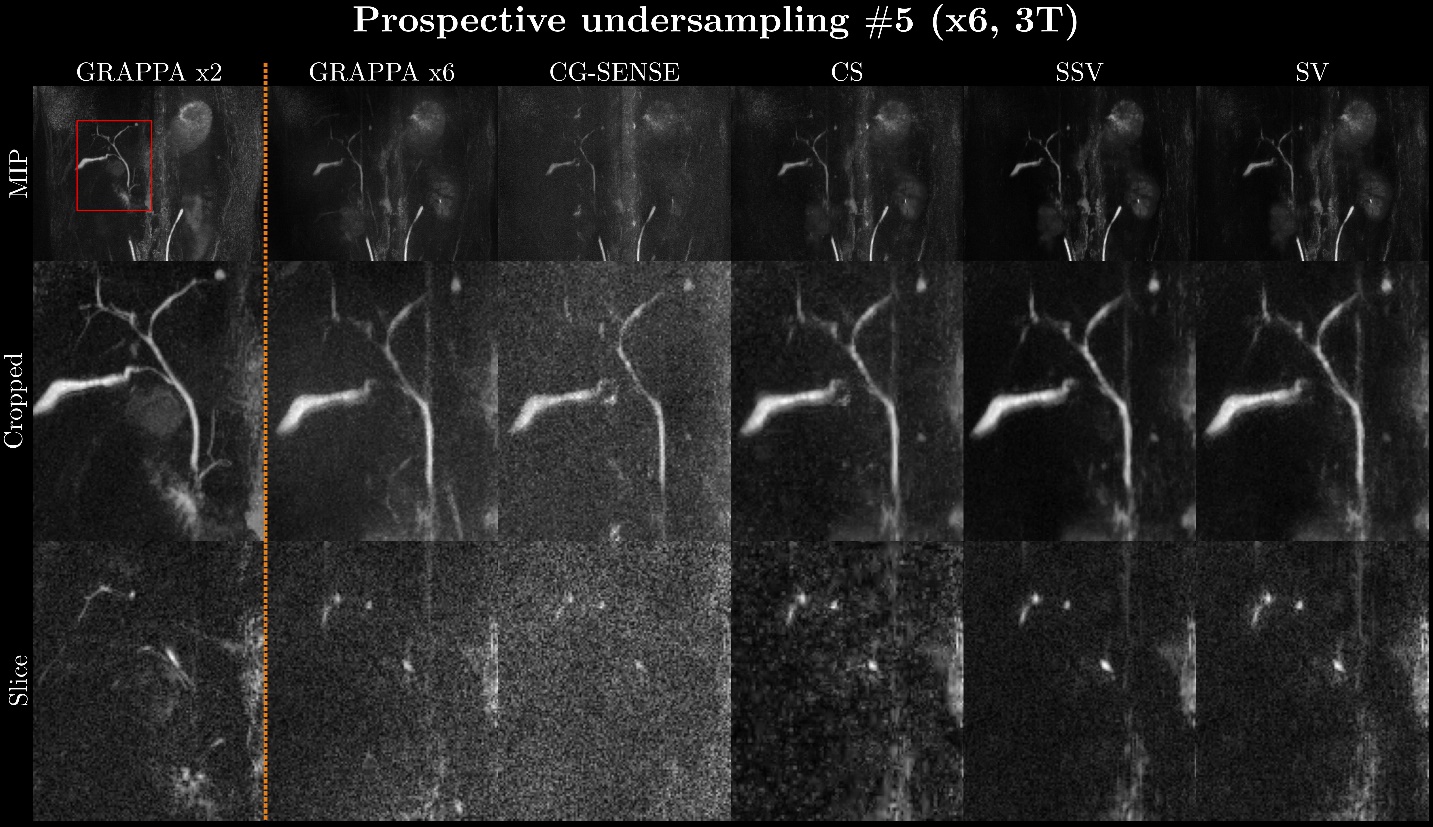
**

**Supplemental Figure 16:** Results of prospective undersampling at 3T for the volunteer #5

**
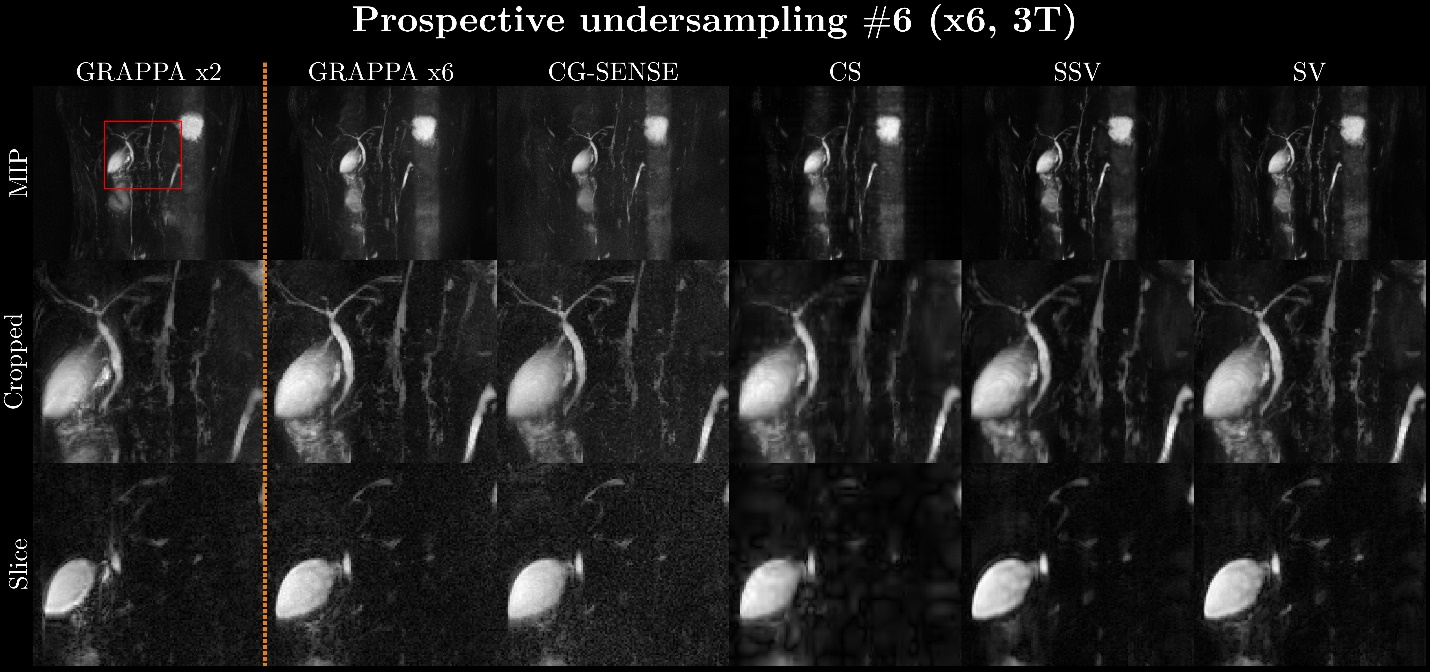
**

**Supplemental Figure 17:** Results of prospective undersampling at 3T for the volunteer #6

**
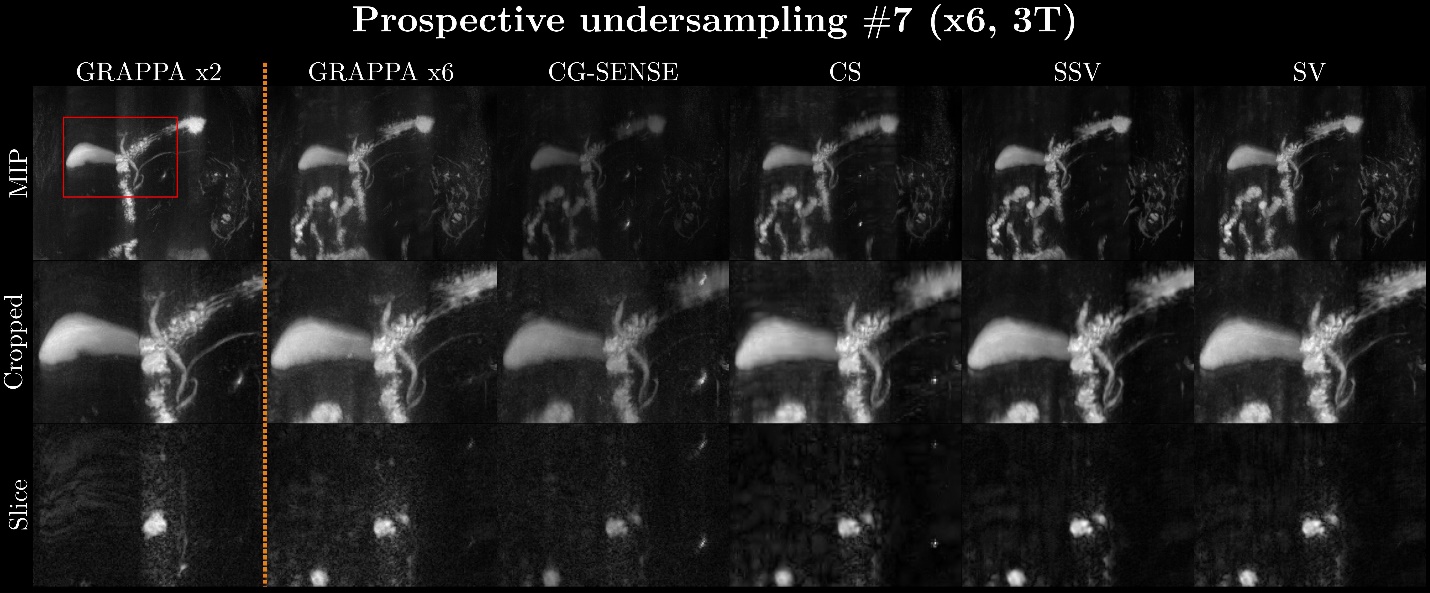
**

**Supplemental Figure 18:** Results of prospective undersampling at 3T for the volunteer #7

**
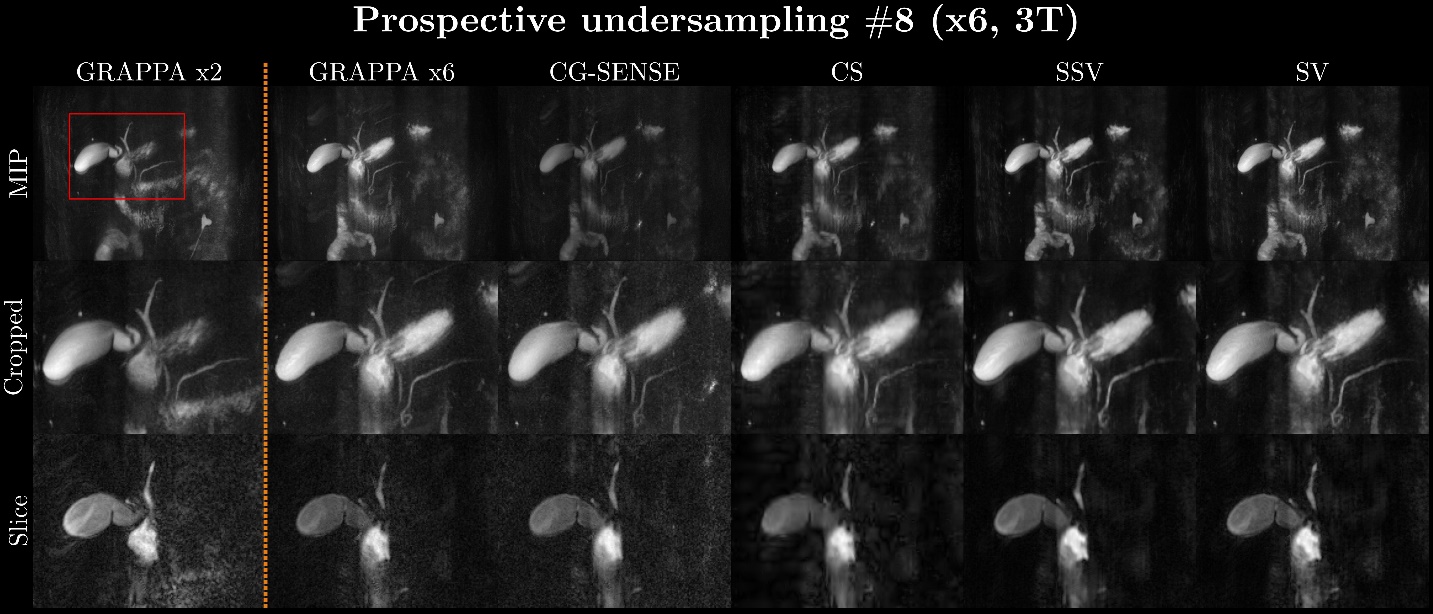
**

**Supplemental Figure 19:** Results of prospective undersampling at 3T for the volunteer #8

**
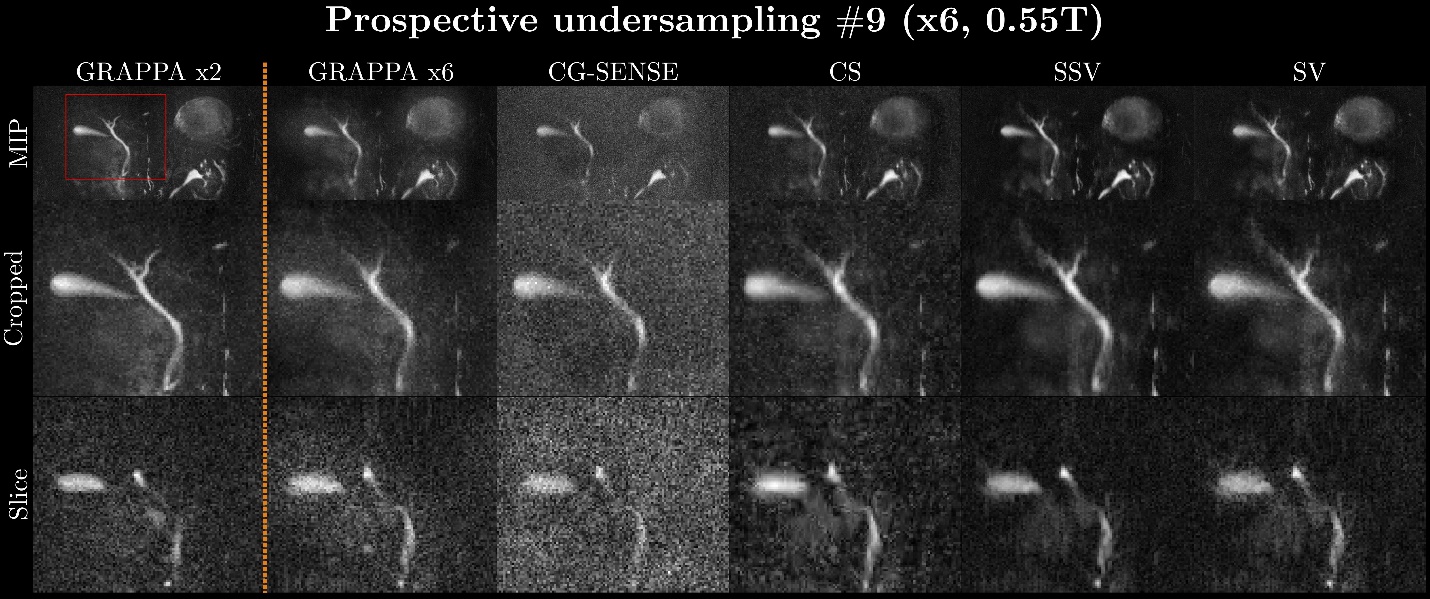
**

**Supplemental Figure 20:** Results of prospective undersampling at 0.55T for the volunteer #9

**
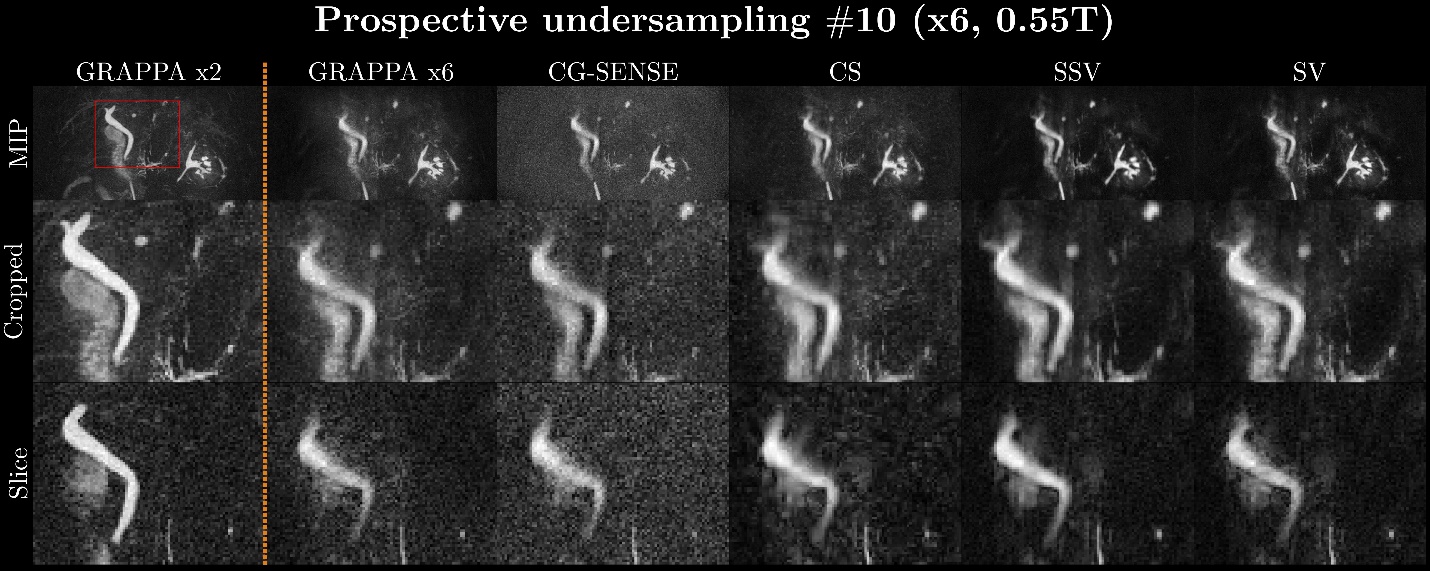
**

**Supplemental Figure 21:** Results of prospective undersampling at 0.55T for the volunteer #10

**
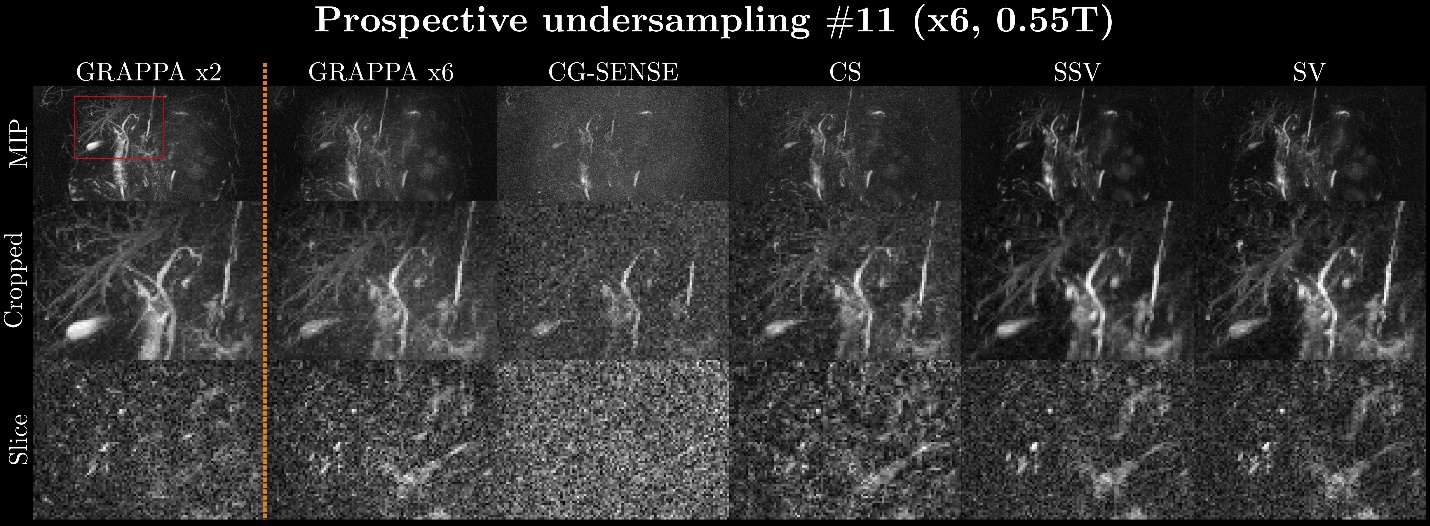
**

**Supplemental Figure 22:** Results of prospective undersampling at 0.55T for the volunteer #11
